# Changes in Children’s Oral Health Related Quality of Life Following Dental Treatment under General Anesthesia

**Published:** 2009

**Authors:** Seyed Ebrahim Jabarifar, Ali Reza Eshghi, Mitra Shabanian, Shahrzad Ahmad

**Affiliations:** * Associate Professor, Department of Pedodontics and Torabinejad Dental Research Center, School of Dentistry, Isfahan University of Medical Sciences, Isfahan, Iran; ** Assistant Professor, Department of Pedodontics and Torabinejad Dental Research Center, School of Dentistry, Isfahan University of Medical Sciences, Isfahan, Iran; *** Assistant Professor, Department of Operative Dentistry and Torabinejad Dental Research Center, School of Dentistry, Isfahan University of Medical Sciences, Isfahan, Iran; **** Dentist, Private Practice, Isfahan University of Medical Sciences, Isfahan, Iran

**Keywords:** Anesthesia, dental care, oral health, quality of life, social impact

## Abstract

**Background:**

Children’s oral health related quality of life (OHRQoL) evaluates the impacts of oral daily activities of children and family on quality of life. Oral health related quality of life as outcome can be used to evaluate the dental health services. This study aimed to assess the extent to which dental treatment under general anesthesia affects quality of life of children and their families.

**Methods:**

One hundred parents of 3-10 year-old children who needed dental treatment under general anesthesia completed a parent-children perception questionnaire (P-CPQ) and family impact scale (FIS) before, and 4 weeks after dental treatment under general anesthesia. The questionnaire had statements related to oral health, functional limitation, emotional state and well being social well-being and family issues. Data were analyzed using SPSS version 11.5.

**Results:**

The mean scores and standard deviations of oral health quality of life of the children before and after dental treatment were 43.3 ± 7.14 and 39.24 ± 5.47 respectively. The mean scores of FIS before and after dental treatment were 8.00 ± 3.21 and 3.66 ± 2.62, respectively. The effect size of mean differences in P-CPQ and FIS scores were 1.84 ± 1.64 and 1.35 ± 4.34, respectively.

**Conclusion:**

Provision of dental treatment under general anesthesia for uncooperative, young children with extensive dental problems had significant effects on quality of life of both children and their families.

## Introduction

When measuring the impact of dental treatment, it is important to assess not only the clinical changes but also the effects of treatment on quality of life. Oral health related quality of life is measured in relation to how the mouth and teeth affect physical, psychological and social well-being and daily activities such as eating, chewing, swallowing, speaking, playing, learning, happiness, embarrassment, and social interactions.^1^–^4^ Measures of quality of life are useful in identifying a wide range of the impacts of childhood illnesses. They can be used to compare dental health care modalities, effectiveness of dental treatments, evaluation of dental health services, and assessing oral health needs.^5^–^7^ A number of valid, reliable generic instruments for measuring children’s oral health quality of life are available including infantile and toddler quality of life (ITQoL)^8^–^12^, child oral impact daily performance (child-OIDP)^13^–^15^, child perception questionnaire (CPQ) for different ages,^16^–^17^ parent-CPQ and early childhood oral health impact scale (ECOHIS) for preschool children and child oral health impact profile (COHIP).^18^–^20^

Dental treatment under general anesthesia is a way to provide a relatively safe treatment for young children and disabled adults with extensive dental problems, who do not accept conventional treat-ment in office setting. A recent study in New Zea-land showed that carrying out dental treatment on children under general anesthesia resulted in significant improvements in the OHRQoL of children.^21^ Therefore, we decided to evaluate the impact of dental treatment on children under general anesthesia in a group of Iranian children and their families using a translated CPQ measure. This study aimed to assess the extent to which dental treatment under general anesthesia changes children’s oral health quality of life and that of their families.

## Methods

Approval was obtained from the Ethics Committee of Isfahan University of Medical Sciences. The study population included a convenience sample of 100 parents of 3-10 year-old children who had dental treatment under general anesthesia in Isfahan Dental School. The children were referred from dental practitioners and community dental clinics because they needed extensive dental treatment and were either very young or uncooperative. They were screened by a pedodontist in the pediatric dentistry department to ascertain the appropriate treatment options and were assessed for uncooperativeness. Children with complex medical conditions such as cerebral palsy, heart failure, Down syndrome and mental retardation were excluded.

We used the translated Persian version of the parent-CPQ (P-CPQ) and family impact scale (FIS).^13^ Two academic faculties of Isfahan Dental School translated the English version of CPQ (parent ver-sion) into the formal Persian language. Parents completed the questionnaire one hour before the treatment of their children. They completed the same questionnaire 4 weeks after dental treatment. The questionnaire had two parts. The first part was the P-CPQ, which assessed oral health, emotional well-being, functional limitations and social well-being using 29 statements with a Likert format re-sponse in children. The second part, the FIS, con-tained statements on impacts of the child’s oral conditions on their families. Sociodemographic information was also recorded. Data were analyzed using SPSS version 11.5. The Cronbach alpha for content validity of P-CPQ and FIS were 0.63 and 0.58, respectively.

## Results

The mean age of the children was 4.85 years. Forty percent of the children were females. Fifty percent of parents had primary school education. Twenty two percent of parents had secondary and 18% had tertiary education level. ANOVA analysis showed a significant relationship between parents’ educa-tion levels and children’s oral health related quality of life and the FIS. An effect size (ES) was a distribution-based measure of changes in CPQ and FIS, pre-treatment and post-treatment measured as follows:

ES = mean (pre-treatment score-post-treatment scores)/SD of pre-treatment scores.

Table 1 presents the data before and after treat-ment. The mean scores of CPQ before and after treatment were 43.03 ± 7.145 and 39.24 ± 5.47 (P < 0.001), respectively. The mean scores of FIS be-fore and after treatment were 8.00 ± 3.21 and 3.66 ± 2.62, respectively (P < 0.0001) (Table 1). The differences of ES average for FIS and CPQ were 1.35 ± 4.34 and 1.84 ± 1.64, respectively (Table 2). There was no significant difference in CPQ or FIS scores between the two genders of children.

**Table 1:** Mean (SD) domain scores of CPQ components at pre and post treatment

Domain	Pre treatment	Post treatment	Difference
Oral health	12.97 (2.44)	5.68 (1.73)	7.29
Functional limitation	9.13 (2.12)	3.45 (1.41)	5.68
Emotional well-being	6.68 (1.39)	2.41 (1.5)	4.27
Social well-being	7.37 (3.86)	3.33 (2.69)	4.04

P < 0.0001

**Table 2:** Mean (SD) of effect size of CPQ domains

Domain	Effect size
Oral health	2.98 (2.11)
Functional limitations	2.67 (1.80)
Emotional well-being	0.63 (1.45)
Social well-being	1.05 (1.20)
Family impact scale	1.35 (4.38)

## Discussion

This research was set out to assess the extent of changes in children’s oral health related quality of life among young children aged 3-10 years with extensive untreated oral problems, who needed oral rehabilitative dental treatment under general anesthesia in a single session. The provision of dental treatment under general anesthesia was as-sociated with significant improvements in children’s oral health related quality of life, irrespec-tive of how much actual treatment they received. All domains of quality of life in children and their families were changed. In a pilot study by Malden *et al*^22^ there were improvements after treatment under general anesthesia in all OHRQoL indica-tors. Parents reported that their children’s quality of life related to eating and sleeping was better than before dental treatment. In a study of Halley White *et al*^21^ parents consistently noted improve-ments in dentally related outcomes after dental treatment under general anesthesia. Parents re-ported that their children’s mastication and sleep-ing have improved following dental treatment. In addition, the social dimensions of quality of life were quite positive; they smiled more frequently, were more attentive in school and more sociable following dental treatment under general anesthe-sia. Their findings are similar to those found in the present study. The Iranian parents perceived that oral functions, emotional well-being and social well-being of children were better after dental treatment. The ES of mean differences of func-tional limitation, emotional well-being, social well-being domains and FIS were 2.98, 2.67, 063, 1.05 and 1.35, respectively.^23^ Although the differences were not significant among ranges of scores, par-ents reported that the changes were noticeable. One of the probable reasons was atypical sample. The difference in ES in oral health was bigger than that in other aspects of children’s quality of life. This was due to short-term evaluation, 4 weeks, after dental treatment. The limitations of this study include parent assessment versus child assessment, no control group and short time follow-up.

## Conclusions

Children’s oral health related quality of life and the impacts on their families were improved signifi-cantly after dental treatment under general anesthe-sia. Oral health related quality of life is a useful outcome for dental treatment assessment. It is rec-ommended to use CPQ instrument in other dental treatments too.
